# The Surgeon-General of the Navy

**Published:** 1888-05

**Authors:** 


The Surgeon-General of the Navy.—Surgeon-General Browne of the navy has entered upon the discharge of his official duties as the head of the medical corps of the navy. The appointment has given marked satisfaction, not only to that corps, but to the medical profession generally. As the director of the *Museum of Hygiene* of the navy, for several years, he did much to make it the great credit that it is to that branch of the service. In all his official relations, in his long career in the navy, the discharge of his duty has been such as to have entitled him to this marked distinction. He enjoys the honor of having been the surgeon of the famous *Kearsarge*, in the battle in which the *Alabama* was sunk off the coast of France

The *Journal and Examiner* tenders to Surgeon-General Browne, who has been one of its corps of collaborators for several years, its congratulations upon his well-merited advancement in his corps.

